# The effect of nurse-led interventions on re-admission and mortality for congestive heart failure: A meta-analysis

**DOI:** 10.1097/MD.0000000000024599

**Published:** 2021-02-19

**Authors:** Xiaoqin Qiu, Chunhan Lan, Jinhua Li, Xi Xiao, Jinlian Li

**Affiliations:** Department of Cardiology, The People's Hospital of Guangxi Zhuang Autonomous Region, Nanning, Guangxi, PR China.

**Keywords:** congestive heart failure, mortality, nurse intervention, nurse-led clinics, readmission, risk ratio

## Abstract

**Background:**

The European Society of Cardiology guidelines recommend the implementation of nurse-led heart failure programs to achieve optimal management of patients with congestive heart failure (CHF). In this analysis, we aimed to systematically show the impact of nurse-led interventions (NLI) on re-admission and mortality in patients with CHF (reduced ejection fraction).

**Methods:**

Publications reporting the impact of NLI on readmission and mortality in patients with CHF were carefully searched from electronic databases. Rehospitalization and mortality were the endpoints. For this analysis, the latest version of the RevMan software was used. Risk ratios (RR) with 95% confidence intervals (CI) were used to represent data following analysis.

**Results:**

A total number of 3282 participants with CHF were included in this analysis. A total of 1571 patients were assigned to the nurse-led intervention group whereas 1711 patients were assigned to the usual care group. The patients had a mean age ranging from 50.8 to 80.3 years. Male patients varied from 27.3% to 73.8%. Comorbidities including hypertension (24.6%–80.0%) and diabetes mellitus (16.7%–59.7%) were also reported. Patients had a mean left ventricular ejection fraction varying from 29.0% to 61.0%. Results of this current analysis showed that rehospitalization (RR: 0.81, 95% CI: 0.74–0.88; *P* = .00001) and mortality (RR: 0.69, 95% CI: 0.56–0.86; *P* = .0009) were significantly lower among CHF patients who were assigned to the nurse-led intervention. Whether during a shorter (3–6 months) or a longer (1–2 years) follow up time period, rehospitalization for shorter [(RR: 0.73, 95% CI: 0.65–0.82; *P* = .00001) vs for longer (RR: 0.81, 95% CI: 0.72–0.91; *P* = .0003) respectively] and mortality for shorter [(RR: 0.55, 95% CI: 0.38–0.80; *P* = .002) vs longer follow up time period (RR: 0.76, 95% CI: 0.58–0.99; *P* = .04) respectively] were significantly lower and in favor of the nurse-led interventional compared to the normal care group.

**Conclusions:**

This systematic review and meta-analysis of randomized controlled trials showed that NLI had significant impacts in reducing the risk of rehospitalization and mortality in these patients with CHF (reduced ejection fraction). Hence, we believe that nurse-led clinics and other interventional programs would be beneficial to patients with heart failure and this practice should, in the future be implemented to the health care system.

## 1 Introduction

Congestive heart failure (CHF) has been an increasing global public health problem and is associated with higher expenditure rate and increased hospital admission as well as mortality.^[1]^ According to a report from the American Heart Association: Heart Disease and Stroke Statistics-2018 Update, CHF affects approximately 6.5 million citizens, with an estimated total number of 1 million new cases diagnosed annually^[2]^ and an estimated cost of $31 billion per year.^[3]^ Also, there is a 2% prevalence of heart failure in the general population and over 10% of those cases are patients aged above 70 years.^[4]^

Today, even if we have improved our health care system and treatment modalities, prognosis for heart failure still remains poor. For example, in the Greece-based heart failure pandemic, representing the clinical and economic burden of heart failure in Greece, the authors demonstrated that despite progress in the management of this chronic disease, about 25% of the patients die during hospitalization, and over 40% are rehospitalized within a period of 1 year.^[5]^ In addition, mortality rate after discharge from the hospital has reached up to 15% and the rate of readmission has reached approximately 20% to 30% within the first month postdischarge.^[6]^ It is now high time to improve the organization of care to provide cost-effective high quality care.

The European Society of Cardiology guidelines recommend the implementation of nurse-led heart failure programs to achieve optimal management of CHF.^[7]^ Even though these programs have shown to reduce readmission, to decrease mortality and to improve the quality of life^[8,9]^ of patients with CHF, it was not easy to implement them in developed countries.

Nurse-led interventions (NLI), carried out by specialized nurses involved giving additional advices, and cares to improve the quality of life of these patients with CHF. To be more precise, nurses provide education on self-care, and they independently perform physical examination as well as assess mental well-being in these patients with CHF.^[10]^ Psychosocial support is also provided by the nurses to the patients and to their families. These nurses also work in close proximity to physicians who can assess and make reasonable and correct decisions about further treatment and management of these patients.

In this analysis, we aimed to systematically show the impact of NLI on readmission and mortality in patients with CHF (with reduced ejection fraction).

## 2 Methods

### 2.1 Data sources and search strategies

Publications reporting the impact of NLI on readmission and mortality in patients with CHF were carefully searched from the following electronic databases:

1. MEDLINE;
2. EMBASE;
3. Web of Science;
4. Cochrane central;
5. http://www.ClinicalTrials.gov;
6. Google scholar;
7. Reference lists of suitable publications.

Only English publications were retrieved for this analysis.

### 2.2 Inclusion and exclusion criteria

Inclusion criteria were as followed:

(a) Only randomized trials based on NLI;
(b) Studies reporting mortality and readmission as their endpoints;
(c) Studies involving participants with CHF;
(d) Studies which were published in English language.

Exclusion criteria were as followed:

(a) Nonrandomized controlled trials, case studies and literature reviews as well as systematic reviews and meta-analyses;
(b) Studies which were not based on NLI;
(c) Studies which did not report mortality or rehospitalization as their endpoints;
(d) Non-English publications;
(e) Duplicated studies.

### 2.3 Outcomes and follow-up

Table 1 shows the types of interventions which were reported, as well as the outcomes and follow-up time periods reported in the original studies.

**Table 1:** Outcomes and follow-up.

Studies	Type of nurse intervention	Outcomes reported	Follow-up time periods
Andryukhin, 2010^[13]^	Nurse-led care program	Mortality and rehospitalization	18 mo
Blue, 2001^[14]^	Specialist nurse intervention	Mortality, rehospitalization	12 mo
Cockayne, 2014^[15]^	Nurse facilitated self- management support	Rehospitalization	12 mo
Cui, 2019^[16]^	Nurse-led structured education program	Readmission, death	12 months
Domingues, 2010^[17]^	Nurse-led education and telephone monitoring	Rehospitalization, death	3 mo
Dunagan, 2005^[18]^	Nurse administered, telephone based disease management	Mortality and readmission	6 and 12 mo
Ekman, 1998^[19]^	Nurse-monitored outpatient care program	Mortality, readmission	6 mo
Emiliane, 2014^[20]^	Nurse-based strategy	Mortality, readmission	6 mo
Ortiz-Bautista, 2018^[21]^	Nurse-led intervention program	Readmission, mortality	24 mo
Pieta, 2006^[22]^	Physician and nurse directed heart failure clinic	Readmission, mortality	12 mo
Riegel, 2002^[23]^	Standardized nurse case-management telephone intervention	Readmission, mortality	3 and 6 mo
Sisk, 2006^[24]^	Nurse-management in minority communities	Rehospitalization	12 mo
Stewart, 2015^[25]^	Nurse-led home based and clinic based secondary prevention program	Rehospitalization. mortality	51 mo
Stromberg, 2003^[26]^	Nurse-led heart failure clinic	Mortality and rehospitalization	3 and 12 mo
You, 2020^[27]^	Nurse-led program of care	Mortality, rehospitalization	3 mo

In this analysis, 2 major outcomes were assessed, namely:

(a) Rehospitalization or readmission, which was defined as the act of being admitted to the hospital again after having recently been discharged from the hospital.
(b) Mortality including death of any cause.

The follow up time period was divided into:

(a) A short term follow-up time period ranging from 3 to 6 months;
(b) A longer follow-up duration ranging from 12 to 24 months.

### 2.4 Data extraction and quality assessment

Data were extracted by 5 independent authors. First of all, the names of authors, the publishing year, and the type of study were retrieved. Secondly, the total number of CHF patients who were randomly assigned to a nurse-led intervention vs a usual care setting was extracted from each study and the total sum was calculated. Data reporting the methodological quality were also extracted for assessment later. In addition, the baseline features including CHF patients’ left ventricular ejection fraction, the comorbidities, the mean age and the respective gender were also extracted. Any disagreement which occurred during this stage of data extraction was thoroughly solved by a careful discussion.

The methodological quality of the randomized trials was assessed based on the criteria suggested by the Cochrane collaboration.^[11]^ Grades (A, B, or C) were allotted signifying low, moderate or high bias risks respectively.

### 2.5 Statistical analysis

For this analysis, the latest version of the RevMan software (version 5.3) was used. Risk ratios (RR) with 95% confidence intervals (CI) were used to represent data following analysis.

The *Q* statistic test was used to assess for heterogeneity. A *P* value less or equal to .05 was considered significant statistically. The I^2^ statistic test was another test which was used to assess for heterogeneity. An increase in heterogeneity was dependent on an increase in the *I*^2^ value.

A fixed statistical effect model was used if a low heterogeneity was present, or else a random effect model was used.

Sensitivity analysis was also carried out. Publication bias was visually observed through funnel plots.

### 2.6 Ethical approval

Ethical approval or any board review approval was not required for this study. This analysis involved data which were extracted from previously published original studies.

## 3 Results

### 3.1 Outcomes of search databases

A total number of 987 articles were obtained through the searched databases (PRISMA guideline).^[12]^

The authors carefully assessed the titles and abstracts, and irrelevant studies were eliminated, resulting in a total number of 95 full text articles which were finally assessed for eligibility.

Another careful assessment was carried out with the full text articles, and studies were eliminated once again based on the following criteria:

1. Nonrandomized studies (n = 14);
2. Review articles (n = 7);
3. Case studies (n = 8);
4. Studies which did not report the required endpoints (n = 9);
5. Studies which were published in a different language (n = 6);
6. Duplicated studies (n = 36).

**Figure 1:**
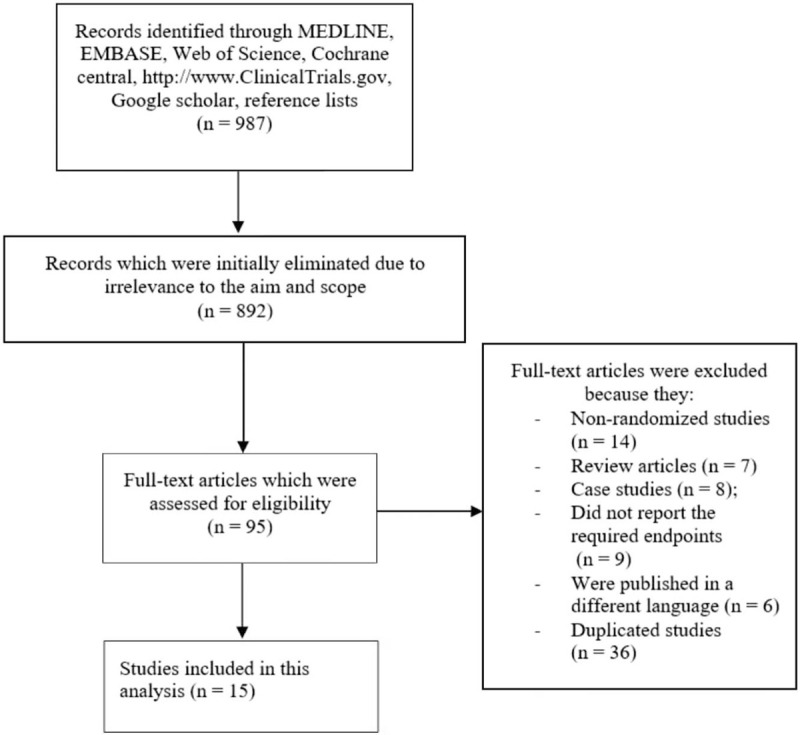
Flow diagram showing study selection.

Finally only 15 studies^[13–27]^ were included in this analysis as shown in Figure 1.

### 3.2 General features of the studies which have been included in this analysis

A total number of 3282 participants with CHF were included in this analysis. A total of 1571 patients were assigned to the nurse-led intervention group whereas 1711 patients were assigned to the usual care setting as shown in Table 2. All the studies were randomized trials.

**Table 2:** General features of the studies.

Studies	Type of study	No of CHF patients in the intervention group (n)	No of CHF patients in the control group (n)	Total No of patients (n)	Bias risk grade
Andryukhin, 2010	RCT	44	41	85	B
Blue, 2001	RCT	84	81	165	B
Cockayne, 2014	RCT	95	165	260	B
Cui, 2019	RCT	48	48	96	B
Domingues, 2010	RCT	48	63	111	B
Dunagan, 2005	RCT	76	75	151	B
Ekman, 1998	RCT	79	79	158	B
Emiliane, 2014	RCT	117	126	243	B
Ortiz-Bautista, 2018	RCT	87	40	127	B
Pieta, 2006	RCT	118	122	240	B
Riegel, 2002	RCT	130	228	358	B
Sisk, 2006	RCT	203	203	406	B
Stewart, 2015	RCT	310	314	624	B
Stromberg, 2003	RCT	52	54	106	B
You, 2020	RCT	80	72	152	B
Total No of participants (n)		1571	1711	3282	

Based on the methodological assessment, a grade “B” was allotted to the randomized trials implying moderate bias risk among all the studies.

### 3.3 Baseline features of the participants

Baseline characteristics of the CHF participants have been listed in Table 3. Left ventricular ejection fraction varied from 29.0% to 61.0%. Comorbidities including hypertension (24.6%–80.0%) and diabetes mellitus (16.7%–59.7%) were also reported. The original studies consisted of male patients varying from 27.3% to 73.8%. The patients had a mean age of 50.8 to 80.3 years as shown in Table 3.

**Table 3:** Baseline features of the participants.

	Age (yrs)	Males (%)	HBP (%)	DM (%)	LVEF (%)
Studies	NI/UC	NI/UC	NI/UC	NI/UC	NI/UC
Andryukhin, 2010	66.5/68.0	27.3/34.2	—	—	—
Blue, 2001	74.4/75.6	64.0/51.0	43.0/52.0	18.0/19.0	—
Cockayne, 2014	70.3/70.8	72.6/72.1	—	-	—
Cui, 2019	55.1/56.6	72.9/68.8	27.1/31.3	20.8/16.7	43.5/42.1
Domingues, 2010	62.0/63.0	67.0/51.0	25.0/33.0	—	29.0/29.0
Dunagan, 2005	70.5/69.4	41.0/47.0	—	—	—
Ekman, 1998	80.3/80.3	58.0/58.0	—	30.0/25.0	—
Emiliane, 2014	62.0/63.0	61.0/64.3	26.0/25.5	-	29.2/30.1
Ortiz-Bautista, 2018	74.0/75.0	69.0/68.0	79.0/80.0	43.0/35.0	36.0/36.0
Pieta, 2006	70.0/71.0	66.0/79.0	39.0/43.0	31.0/28.0	30.6/31.3
Riegel, 2002	72.5/74.6	53.8/46.1	24.6/42.0	43.1/41.6	—
Sisk, 2006	59.6/59.3	55.2/52.2	70.0/71.4	36.5/39.9	—
Stewart, 2015	66.0/66.0	68.0/73.0	60.0/63.0	26.0/26.0	60.0/61.0
Stromberg, 2003	77.0/78.0	63.5/59.3	50.0/29.6	15.4/31.5	—
You, 2020	50.8/51.3	73.8/73.6	71.3/72.2	58.8/59.7	39.0/39.4

### 3.4 Rehospitalization and mortality

**Figure 2:**
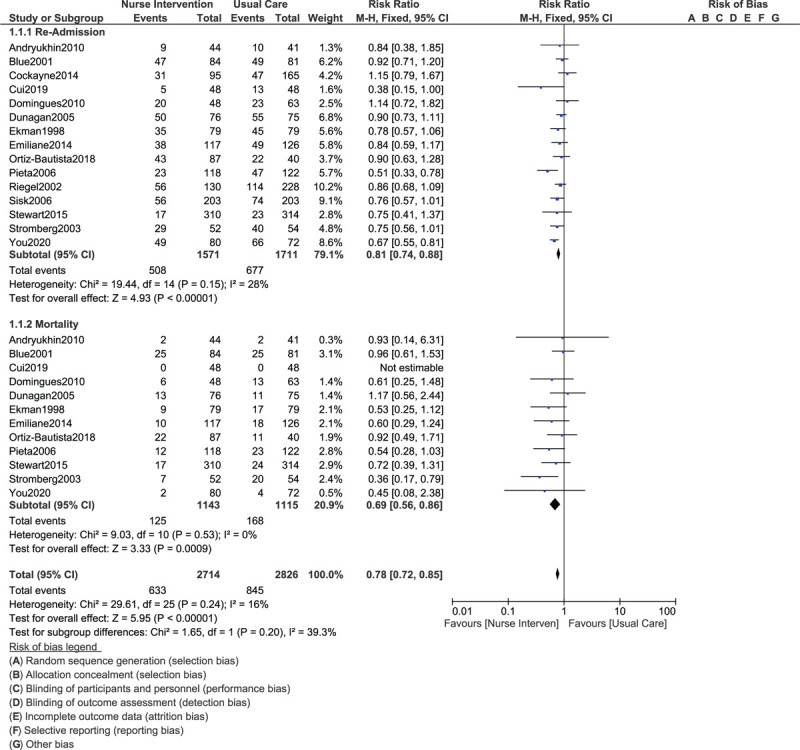
Comparing rehospitalization and mortality in congestive heart failure patients assigned to a nurse-led intervention program vs a control group.

Results of this current analysis showed that rehospitalization (RR: 0.81, 95% CI: 0.74–0.88; *P* = .00001) and mortality (RR: 0.69, 95% CI: 0.56–0.86; *P* = .0009) were significantly lower among CHF patients who were assigned to the nurse-led intervention as shown in Figure 2.

**Figure 3:**
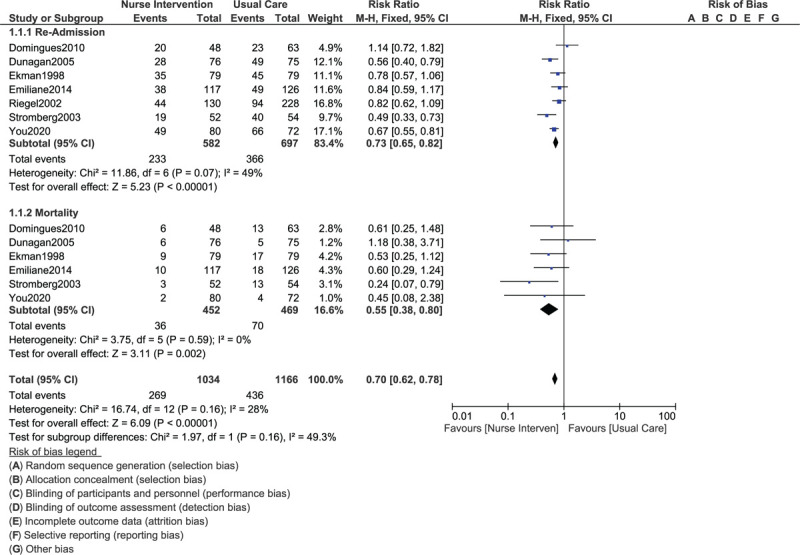
Comparing rehospitalization and mortality in congestive heart failure patients assigned to a nurse-led intervention program vs a control group during a short-term follow up (3–6 months).

Even during a shorter follow-up time period of 3 to 6 months, rehospitalization (RR: 0.73, 95% CI: 0.65–0.82; *P* = .00001) and mortality (RR: 0.55, 95% CI: 0.38–0.80; *P* = .002) were significantly lower in the nurse-led interventional group when compared to the usual care setting as shown in Figure 3.

**Figure 4:**
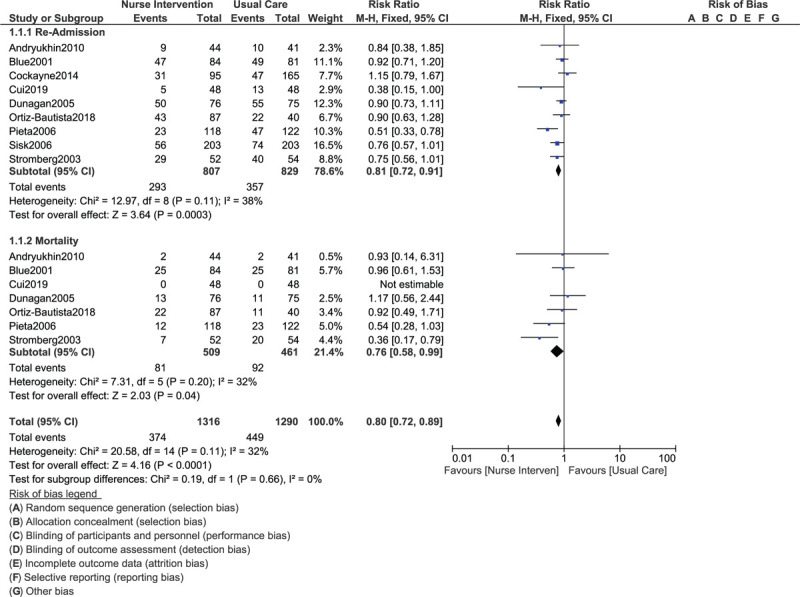
Comparing rehospitalization and mortality in congestive heart failure patients assigned to a nurse-led intervention program vs a control group during a long-term follow up (1–2 years).

During a longer follow-up time period of 12 to 24 months, rehospitalization (RR: 0.81, 95% CI: 0.72–0.91; *P* = .0003) and mortality (RR: 0.76, 95% CI: 0.58–0.99; *P* = .04) were still significantly in favor of the nurse-led intervention as shown in Figure 4.

### 3.5 Sensitivity analysis and publication bias

**Figure 5:**
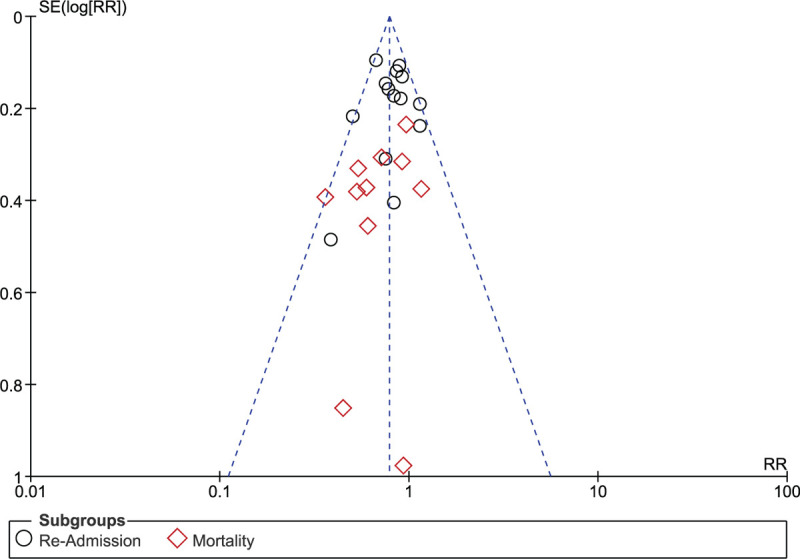
Funnel plot showing publication bias.

Sensitivity analysis was carried out. Each time the new result was compared with previous ones to observe for any significant deviation from the main results. However, consistent results were obtained all over. In addition, a funnel plot was generated through RevMan to visually observe publication bias as shown in Figure 5.

## 4 Discussion

With new development in the medical line, several new techniques to diagnose and manage heart failure have been introduced. Nowadays, multidirectional strain parameters derived from three-dimensional echocardiography for predicting left ventricular remodeling after myocardial infarction are being used.^[28]^ Newer cardiac assisted devices for decompensated heart failure have also been introduced.^[29]^ The magnetic levitated centrifugal continuous flow circulatory pump and the axial continuous flow pump for advanced heart failure have also been developed.^[30]^ However, mortality rate due to heart failure, and the rate of readmission for the same chronic disease have still not decreased. The European Society of Cardiology guidelines recommend the implementation of nurse-led heart failure programs to achieve optimal management of CHF.^[7]^

In this current analysis, the authors demonstrated that NLI were associated with significantly lower rehospitalization and mortality in patients with CHF (with reduced ejection fraction) whether during a short or long term follow up time periods.

To support the results of this analysis, a recent retrospective review including 413 patients with decompensated heart failure showed that a nurse-led heart failure program was independently associated with an increased survival rate among these patients,^[31]^ supporting the results of this analysis. Another Hong Kong based study further supported the result of our current analysis showing that in a nurse-led heart failure clinic whereby interventions were carried out by nurses,^[32]^ mortality rate and rehospitalization were significantly decreased in comparison to patients who were not intervened by these nurses.

In addition, in a multisite implementation study which showed the evaluation of a nurse practitioner disease management model for CHF, it was discussed that there was less admission due to CHF or any other cause in the nurse-led intervention group at 1 year.^[33]^ It was shown that this improved outcome was most probably due to the care and coordination which were provided by the nurse practitioners. They carefully monitored comorbidities such as diabetes, hypertension, and coronary artery disease which were directly linked to heart failure. It was also suggested that nurses would motivate and educate patients to change their life habits, referred patients to relevant specialities which were concerned when required, and would motivate them to maintain medication compliance in order to reduce acute exacerbation of their CHF and other chronic diseases. All these would partly be associated with reduced readmission to hospitals.

Also, the specialized nurses in nurse-led clinics were responsible for the prescription and titration of heart failure medications.^[33]^ They were familiar with the current heart failure guidelines and would review their patients on a regular basis and were specialized to only treat heart failure patients.

At last, a systematic review and meta-analysis highlighted the potential ability of heart failure management programs which were carried out by nurse intervention predischarge to reduce hospital readmission.^[34]^ Our present analysis, which additionally assessed mortality and rehospitalization during a shorter and longer follow up time period respectively, is in favor of a nurse-led intervention program for the management of patients with CHF. Implementation of this practice might be cost effective and beneficial in terms of minimizing readmission, and prolonging survival rate in these patients.

## 5 Limitations

The types of nursing interventions were not similar in all of the original studies. Even though all the original studies involved NLI, minor differences in their approach might contribute to the limitation of this analysis. Another limitation might be due to the fact that the follow-up time periods were not similar in all of the studies. Moreover, since in this analysis we could not assess the causes of mortality of the patients with CHF, this might be considered as another limitation of this analysis. In addition, the duration of this chronic disease was not reported in many of the original studies. Also, we have ignored the medications which could have had an impact in reducing readmission and mortality in these patients. At last, all the trials which were included in this analysis involved heart failure patients with reduced ejection fraction except for one study which consisted of heart failure participants with preserved ejection fraction. However, because the particular trial only consisted of a minor number of participants, even if it has been included in this analysis, it would not have any major impact on the outcomes.

## 6 Conclusions

This systematic review and meta-analysis of randomized controlled trials showed that NLI had significant impacts in reducing the risk of rehospitalization and mortality in these patients with CHF (with reduced ejection fraction). Hence, we believe that nurse-led clinics and other interventional programs would be beneficial to patients with heart failure and this practice should be implemented to the health care system.

## Author contributions

**Conceptualization:** Xiaoqin Qiu, Chunhan Lan, Jinhua Li, Xi Xiao, Jinlian Li.

**Data curation:** Xiaoqin Qiu, Chunhan Lan, Jinhua Li, Xi Xiao, Jinlian Li.

**Formal analysis:** Xiaoqin Qiu, Chunhan Lan, Jinhua Li, Xi Xiao, Jinlian Li.

**Funding acquisition:** Xiaoqin Qiu, Chunhan Lan, Jinhua Li, Xi Xiao, Jinlian Li.

**Investigation:** Xiaoqin Qiu, Chunhan Lan, Jinhua Li, Xi Xiao, Jinlian Li.

**Methodology:** Xiaoqin Qiu, Chunhan Lan, Jinhua Li, Xi Xiao, Jinlian Li.

**Project administration:** Xiaoqin Qiu, Chunhan Lan, Jinhua Li, Xi Xiao, Jinlian Li.

**Resources:** Xiaoqin Qiu, Chunhan Lan, Jinhua Li, Xi Xiao, Jinlian Li.

**Software:** Xiaoqin Qiu, Chunhan Lan, Jinhua Li, Xi Xiao, Jinlian Li.

**Supervision:** Xiaoqin Qiu, Chunhan Lan, Jinhua Li, Xi Xiao, Jinlian Li.

**Validation:** Xiaoqin Qiu, Chunhan Lan, Jinhua Li, Xi Xiao, Jinlian Li.

**Visualization:** Xiaoqin Qiu, Chunhan Lan, Jinhua Li, Xi Xiao, Jinlian Li.

**Writing – original draft:** Xiaoqin Qiu, Chunhan Lan, Jinhua Li, Xi Xiao, Jinlian Li.

**Writing – review & editing:** Xiaoqin Qiu, Chunhan Lan, Jinhua Li, Xi Xiao, Jinlian Li.

## References

[1] Laribi S Aouba A Nikolaou M. GREAT network. Trends in death attributed to heart failure over the past two decades in Europe. Eur J Heart Fail 2012;14:234–9. 22237388 10.1093/eurjhf/hfr182

[2] Benjamin EJ Virani SS Callaway CW. Heart disease and stroke statistics-2018 update: a report from the American Heart Association. Circulation 2018;137:e67–492. 29386200 10.1161/CIR.0000000000000558

[3] Mozaffarian D Benjamin EJ Go AS. Heart disease and stroke statistics-2016 update: a report from the American Heart Association. Circulation 2016;133:e38–60. 26673558 10.1161/CIR.0000000000000350

[4] Savarese G Lund LH. Global public health burden of heart failure. Card Fail Rev 2017;3:7–11. 28785469 10.15420/cfr.2016:25:2 PMC5494150

[5] Stafylas P Farmakis D Kourlaba G. The heart failure pandemic: the clinical and economic burden in Greece. Int J Cardiol 2017;227:923–9. 27915082 10.1016/j.ijcard.2016.10.042

[6] Greene SJ Fonarow GC Vaduganathan M. The vulnerable phase after hospitalization for heart failure. Nat Rev Cardiol 2015;12:220–9. 25666406 10.1038/nrcardio.2015.14

[7] McMurray JJ Adamopoulos S Anker SD. ESC Committee for Practice Guidelines. ESC Guidelines for the diagnosis and treatment of acute and chronic heart failure 2012: The Task Force for the Diagnosis and Treatment of Acute and Chronic Heart Failure 2012 of the European Society of Cardiology. Developed in collaboration with the Heart Failure Association (HFA) of the ESC. Eur Heart J 2012;33:1787–847. 22611136 10.1093/eurheartj/ehs104

[8] Phillips CO Singa RM Rubin HR. Complexity of program and clinical outcomes of heart failure disease management incorporating specialist nurse-led heart failure clinics. A meta-regression analysis. Eur J Heart Fail 2005;7:333–41. 15718173 10.1016/j.ejheart.2005.01.011

[9] Thompson DR Roebuck A Stewart S. Effects of a nurse-led, clinic and home-based intervention on recurrent hospital use in chronic heart failure. Eur J Heart Fail 2005;7:377–84. 15718178 10.1016/j.ejheart.2004.10.008

[10] Riley JP Astin F Crespo-Leiro MG. Heart Failure Association of the European Society of Cardiology heart failure nurse curriculum. Eur J Heart Fail 2016;18:736–43. 27220672 10.1002/ejhf.568

[11] Higgins JP Altman DG Gøtzsche PC. Cochrane Bias Methods Group; Cochrane Statistical Methods Group.The Cochrane Collaboration's tool for assessing risk of bias in randomised trials. BMJ 2011;343:d5928. 22008217 10.1136/bmj.d5928 PMC3196245

[12] Liberati A Altman DG Tetzlaff J. The PRISMA statement for reporting systematic reviews and meta-analyses of studies that evaluate healthcare interventions: explanation and elaboration. BMJ 2009;339:b2700. 19622552 10.1136/bmj.b2700 PMC2714672

[13] Andryukhin A Frolova E Vaes B. The impact of a nurse-led care programme on events and physical and psychosocial parameters in patients with heart failure with preserved ejection fraction: a randomized clinical trial in primary care in Russia. Eur J Gen Pract 2010;16:205–14. 21073267 10.3109/13814788.2010.527938

[14] Blue L Lang E McMurray JJ. Randomised controlled trial of specialist nurse intervention in heart failure. BMJ 2001;323:715–8. 11576977 10.1136/bmj.323.7315.715 PMC56888

[15] Cockayne S Pattenden J Worthy G. Nurse facilitated Self-management support for people with heart failure and their family carers (SEMAPHFOR): a randomised controlled trial. Int J Nurs Stud 2014;51:1207–13. 24508285 10.1016/j.ijnurstu.2014.01.010

[16] Cui X Zhou X Ma LL. A nurse-led structured education program improves self-management skills and reduces hospital readmissions in patients with chronic heart failure: a randomized and controlled trial in China. Rural Remote Health 2019;19:5270. 31113205 10.22605/RRH5270

[17] Domingues FB Clausell N Aliti GB. Education and telephone monitoring by nurses of patients with heart failure: randomized clinical trial. Arq Bras Cardiol 2011;96:233–9. 21308343 10.1590/s0066-782x2011005000014

[18] Dunagan WC Littenberg B Ewald GA. Randomized trial of a nurse-administered, telephone-based disease management program for patients with heart failure. J Card Fail 2005;11:358–65. 15948086 10.1016/j.cardfail.2004.12.004

[19] Ekman I Andersson B Ehnfors M. Feasibility of a nurse-monitored, outpatient-care programme for elderly patients with moderate-to-severe, chronic heart failure. Eur Heart J 1998;19:1254–60. 9740348 10.1053/euhj.1998.1095

[20] de Souza EN Rohde LE Ruschel KB. A nurse-based strategy reduces heart failure morbidity in patients admitted for acute decompensated heart failure in Brazil: the HELEN-II clinical trial. Eur J Heart Fail 2014;16:1002–8. 25044072 10.1002/ejhf.125

[21] Ortiz-Bautista C Morán-Fernández L Díaz-García M. Evaluation of a nurse-led intervention program in heart failure: a randomized trial. Med Clin (Barc) 2019;152:431–7. 30314739 10.1016/j.medcli.2018.08.005

[22] de la Porte PW Lok DJ van Veldhuisen DJ. Added value of a physician-and-nurse-directed heart failure clinic: results from the Deventer-Alkmaar heart failure study. Heart 2007;93:819–25. 17065182 10.1136/hrt.2006.095810 PMC1994472

[23] Riegel B Carlson B Kopp Z. Effect of a standardized nurse case-management telephone intervention on resource use in patients with chronic heart failure. Arch Intern Med 2002;162:705–12. 11911726 10.1001/archinte.162.6.705

[24] Sisk JE Hebert PL Horowitz CR. Effects of nurse management on the quality of heart failure care in minority communities: a randomized trial. Ann Intern Med 2006;145:273–83. 16908918 10.7326/0003-4819-145-4-200608150-00007 PMC4307780

[25] Stewart S Chan YK Wong C. NIL-CHF Investigators. Impact of a nurse-led home and clinic-based secondary prevention programme to prevent progressive cardiac dysfunction in high-risk individuals: the Nurse-led Intervention for Less Chronic Heart Failure (NIL-CHF) randomized controlled study. Eur J Heart Fail 2015;17:620–30. 25899895 10.1002/ejhf.272

[26] Strömberg A Mårtensson J Fridlund B. Nurse-led heart failure clinics improve survival and self-care behaviour in patients with heart failure: results from a prospective, randomised trial. Eur Heart J 2003;24:1014–23. 12788301 10.1016/s0195-668x(03)00112-x

[27] You J Wang S Li J. Usefulness of a nurse-led program of care for management of patients with chronic heart failure. Med Sci Monit 2020;26:e920469. 32068197 10.12659/MSM.920469 PMC7047924

[28] Xu L Huang X Ma J. Value of three-dimensional strain parameters for predicting left ventricular remodeling after ST-elevation myocardial infarction. Int J Cardiovasc Imaging 2017;33:663–73. 28150084 10.1007/s10554-016-1053-3

[29] Mishra S. Upscaling cardiac assist devices in decompensated heart failure: choice of device and its timing. Indian Heart J 2016;68: (Suppl 1): S1–4. 10.1016/j.ihj.2015.12.012 PMC4824335 27056646

[30] Tang B Yang H. Two year adverse outcomes of the magnetic levitated centrifugal continuous flow circulatory pump versus the axial continuous-flow pump for advanced heart failure: a systematic review and meta-analysis of randomized controlled trials. Medicine (Baltimore) 2020;99:e19393. 32118791 10.1097/MD.0000000000019393 PMC7478487

[31] Bdeir B Conboy T Mukhtar A. Impact of a nurse-led heart failure program on all-cause mortality. J Cardiovasc Nurs 2015;30:E7–14. 24496326 10.1097/JCN.0000000000000133

[32] Cheng HY Chair SY Wang Q. Effects of a nurse-led heart failure clinic on hospital readmission and mortality in Hong Kong. J Geriatr Cardiol 2016;13:415–9. 27594868 10.11909/j.issn.1671-5411.2016.05.013 PMC4984577

[33] Lowery J Hopp F Subramanian U. Evaluation of a nurse practitioner disease management model for chronic heart failure: a multi-site implementation study. Congest Heart Fail 2012;18:64–71. 22277180 10.1111/j.1751-7133.2011.00228.x

[34] Lambrinou E Kalogirou F Lamnisos D. Effectiveness of heart failure management programmes with nurse-led discharge planning in reducing re-admissions: a systematic review and meta-analysis. Int J Nurs Stud 2012;49:610–24. 22196054 10.1016/j.ijnurstu.2011.11.002

